# Uniformity and passivation research of Al_2_O_3_ film on silicon substrate prepared by plasma-enhanced atom layer deposition

**DOI:** 10.1186/s11671-015-0831-5

**Published:** 2015-03-13

**Authors:** Endong Jia, Chunlan Zhou, Wenjing Wang

**Affiliations:** Institute Of Electrical Engineering, Key Laboratory of Solar Thermal Energy and Photovoltaic System, Chinese Academy of Sciences, No.6 Bertiao, Zhongguancun, Beijing, 100190 China

**Keywords:** PEALD, Al_2_O_3_, Silicon surface passivation, Uniformity

## Abstract

Plasma-enhanced atom layer deposition (PEALD) can deposit denser films than those prepared by thermal ALD. But the improvement on thickness uniformity and the decrease of defect density of the films deposited by PEALD need further research. A PEALD process from trimethyl-aluminum (TMA) and oxygen plasma was investigated to study the influence of the conditions with different plasma powers and deposition temperatures on uniformity and growth rate. The thickness and refractive index of films were measured by ellipsometry, and the passivation effect of alumina on n-type silicon before and after annealing was measured by microwave photoconductivity decay method. Also, the effects of deposition temperature and annealing temperature on effective minority carrier lifetime were investigated. Capacitance-voltage and conductance-voltage measurements were used to investigate the interface defect density of state (*D*_it_) of Al_2_O_3_/Si. Finally, Al diffusion P^+^ emitter on n-type silicon was passivated by PEALD Al_2_O_3_ films. The conclusion is that the condition of lower substrate temperature accelerates the growth of films and that the condition of lower plasma power controls the films’ uniformity. The annealing temperature is higher for samples prepared at lower substrate temperature in order to get the better surface passivation effects. Heavier doping concentration of Al increased passivation quality after annealing by the effective minority carrier lifetime up to 100 μs.

## Background

As the crystalline silicon solar cell industry matures, minority carrier lifetime increases and the thickness of wafer decreases, causing front-surface passivation quality approaching the limit of the theoretical calculation. While the back of the solar cell is Al back-surface field (Al-BSF), surface recombination increases significantly. To decay back-surface minority carrier recombination is a major solution in all to increase incident photon-to-electron conversion efficiency (IPCE). Al_2_O_3_ deposition on back surface was proven to be effective on passivation. The result of simulation and calculation by PC1D concludes that using alumina to passivate back surface could increase IPCE of c-Si solar cell over 1% [1]. The preparation process of Al_2_O_3_ can be classified to atom layer deposition (ALD), PECVD, and APCVD. Considering the passivation quality for better film with high uniformity and less defect density, ALD method is the most appropriate. Two major ALD methods are thermal ALD and plasma-enhanced ALD to prepare Al_2_O_3_ films. Comparing to thermal ALD, plasma-enhanced atom layer deposition (PEALD) allows deposition at significantly lower temperature with better film properties. And more, the method saves more energy and is more suitable for industrialization [2].

Passivation quality of Al_2_O_3_ is contributed by both field-effect passivation and chemical passivation. The fixed interface charge of alumina prepared by PEALD is negative in the amount of approximately 1 × 10^13^ cm^−2^ [3], to push the electrons away from the surface and suitable for p-type silicon passivation.

The effects of PEALD process parameters including plasma power, deposition temperature (substrate temperature), and annealing temperature on passivation quality were investigated. The dependence of Al doping concentration of Al diffusion P^+^ emitter based on n-type silicon substrate in passivation quality was investigated. Al_2_O_3_ film character was determined by evaluation of thickness uniformity and growth rate measured by ellipsometry. Surface passivation quality was determined by evaluation of effective minority carrier lifetime and surface fixed charge *Q*_f_ in microwave photoconductivity decay method.

## Methods

The depositions were carried out on 4′′ n-type Cz, Fz silicon samples with thickness of about 500 μm. Square resistance varied with the change of Al doping concentration. To get different Al doping concentrations, silicon samples were screen printed with aluminum paste on two sides firstly, then were fired in sintering furnace and were treated with hydrochloric acid to remove Al paste and alloy layers, and lastly were etched by KOH (10% in mass concentration, 80°C) at different times. Chemical treatments prior to PEALD deposition were proceeded in sulphuric acid mixture with hydrogen peroxide (H_2_SO_4_:H_2_O_2_ = 4:1) step and hydrofluoric acid (volume fraction 1%) step, separately. Samples were cleaned in sulphuric acid mixture with hydrogen peroxide at 110°C for 10 min to oxidate and remove organic impurities on surface and also to dissolve metal ions from surface to solution. Then, samples were treated in hydrofluoric acid for 1 min at room temperature to remove silicon oxide which is 2 to 4 nm [4]. At last, samples were capped with PEALD Al_2_O_3_ using Al(CH_3_)_3_ (TMA) and plasma O as reactants. The deposition of 200 cycles resulted in a thickness of about 20 nm. Thickness and uniformity were determined by process parameter: plasma powers (45 and 80 W), at this time, deposition temperature was 200°C. Growth per cycle (GPC) was inspected when changing deposition temperature to 100°C, 200°C, 250°C, and 300°C while the plasma power kept 80 W. Passivation quality was determined by the parameters of deposition temperature changing from 100°C to 300°C, annealing temperature changing from 325°C to 500°C, firing temperature about 800°C, also doping concentration of Al, which was evaluated by square resistance. Annealing steps lasted for 10 min, and samples were annealed at ambient air.

Average thickness and uniformity of Al_2_O_3_ film were measured by ellipsometry method. Further, GPC was calculated by the total thickness divided by the cycle counts. The surface passivation quality was determined by the evaluation of the effective minority carrier lifetime *τ*_eff_ using photo conductance decay measurements with a Semilab WT-2000 instrument (Semilab, Budapest, Hungary). The field-effect passivation was determined by the surface fixed negative charge *Q*_f_ measured by the method of charge injection to decay the effective minority carrier lifetime on Corona probe (Semilab, Budapest, Hungary) technique, the amount of injection charge (positive) was equal to that of *Q*_f_ when lifetime decayed most. Capacitance-voltage and conductance-voltage measurements were used to investigate the interface defect density of state (*D*_it_) of Al_2_O_3_/Si. Metal-oxide-semiconductor capacitors were prepared for electrical testing of the Al_2_O_3_ films. Aluminum dots with variable area were evaporated on the surface of the Al_2_O_3_ using a shadow mask. The back contacts were achieved by evaporating a uniform aluminum layer on the silicon substrate.

## Results and discussion

### Growth rate/uniformity

**Figure 1:**
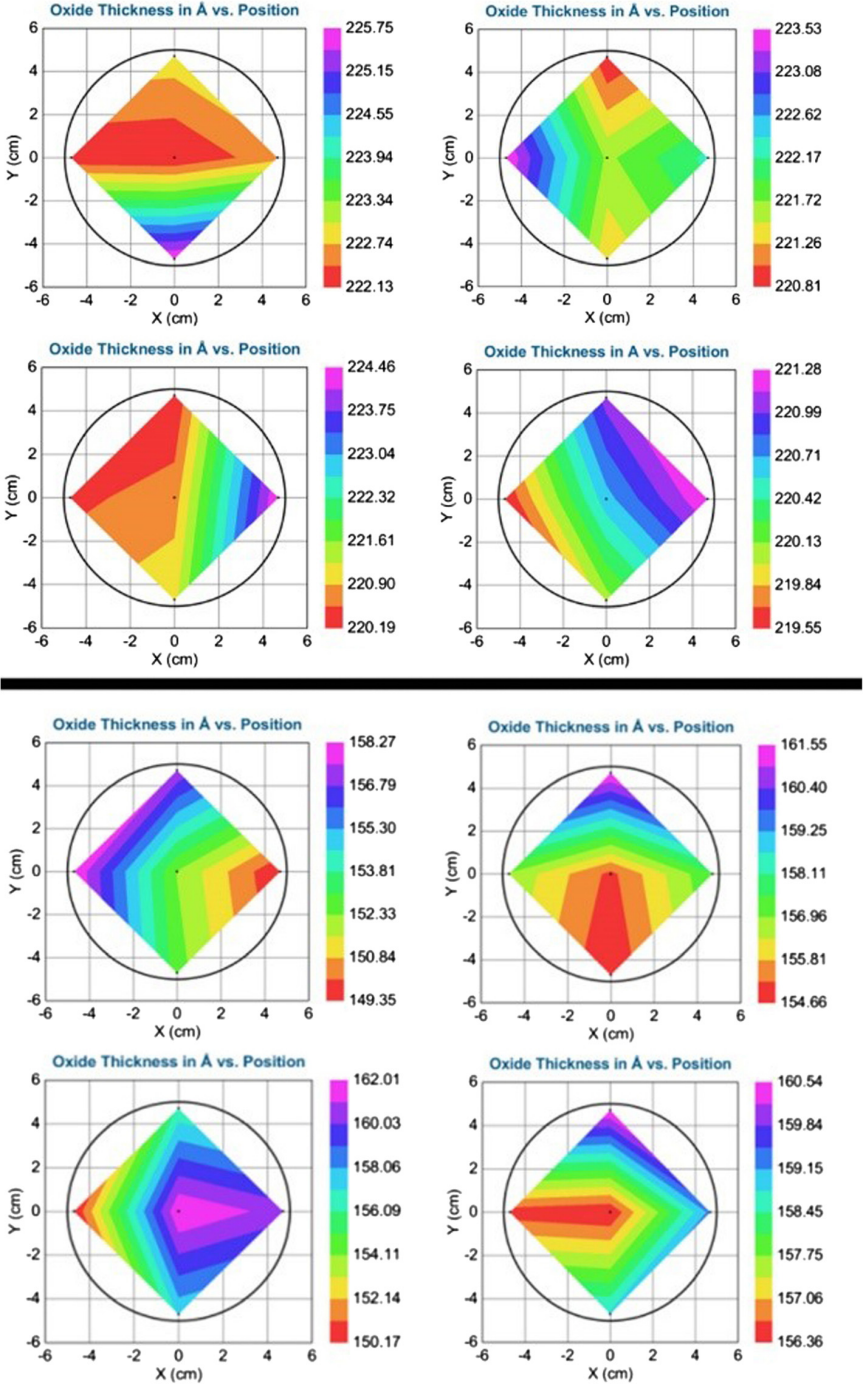
**2D plots of thickness variation of PEALD alumina on 4′′ silicon samples, deposition temperature 200** **°C** **.** Top four on plasma power 45 W, 200 cycles; bottom four on plasma power 80 W, 120 cycles.

Figure 1 shows the color contour plots of PEALD Al_2_O_3_ thickness on 4′′ silicon substrates. On the condition of plasma power 45 W and cycle number 200, thickness varied from approximately 22 to approximately 22.5 nm and diversity between four samples was little. But when plasma power increased to 80 W (cycle number 120), differences of thickness not only between four samples but also in one sample were more, thickness varied from approximately 15 to approximately 16 nm. Uniformity becomes worse on 80 W plasma power than on 45 W. As shown in Table 1 (data are disposed from Figure 1), uniformity of PEALD Al_2_O_3_ was excellent for the average standard deviation not over 2%. Uniformity of Al_2_O_3_ was even better when deposited on 45 W plasma power than that deposited on 80 W plasma power. One possible reason is parasitic CVD occurred when PEALD Al_2_O_3_ plasma power was 80 W. When plasma power is higher, oxygen plasma on the deposited surface have larger kinetic to promote deposition speed, but plasma irradiation would also do damage to the deposited surface and cause worse uniformity. Therefore, Al_2_O_3_ deposition GPC is larger on 80 W than that on 45 W, but uniformity is worse on 80 W than that on 45 W. According to the reaction Equation 1 [5], lower plasma power led less active oxygen plasma to promote Al_2_O_3_ deposition.

**Table 1:** **Thickness deviation and GPC vary on different plasma powers**

**Plasma power (W)**	**Standard deviation**	**GPC (** **nm/cycle** **)**
80	0.353 nm (2.0%)	0.131
45	0.155 nm (0.7%)	0.111

1 $$ \mathrm{A}\mathrm{l}\mathrm{O}-{\mathrm{AlCH}}_3^{*}+\mathrm{O}(g)\to \mathrm{Al}\mathrm{O}\mathrm{Al}-{\mathrm{OH}}^{*}+{\mathrm{H}}_2\mathrm{O}(g)+{\mathrm{CO}}_x(g) $$

**Figure 2:**
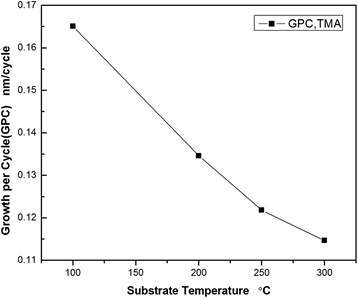
**Growth rate varies at different substrate temperatures (deposition temperature) on the condition that plasma power is 80 W.**

GPC decayed when deposition temperature increased, from 0.165 to 0.123 nm/cycle as shown in Figure 2, which could be explained by ‘ALD process window’. The growth rate decayed with deposition temperature increase was due to the reduction in adsorption site density on the depositing surface for adsorption desorption. Deposition temperature increasing caused adsorption molecules to have enough kinetic energy over adsorption energy to escape from surface, which decayed the Al_2_O_3_ films thickness.

### Deposition and annealing temperature/passivation quality

**Figure 3:**
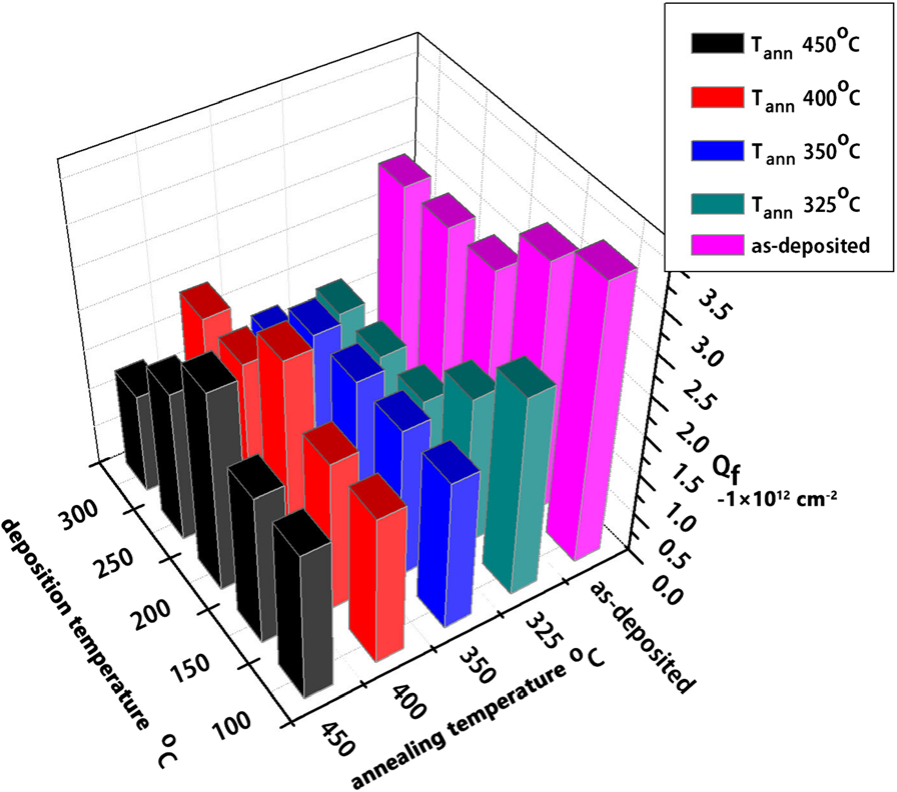
**Surface fixed charge density at different deposition temperatures and annealing temperatures.** In units of −1 × 10^12^ cm^−2^ (in air) and measured by Corona Probe method.

Figure 3 shows the surface fixed charge density as a function of field-effect passivation at various deposition temperatures before and after annealing at variable temperature. Surface fixed charge density of PEALD Al_2_O_3_ (*Q*_f_) reduced with the increasing of annealing temperature when the deposition temperature was below 150°C. When the deposition temperature was over 150°C and below 300°C, *Q*_f_ descended and then got the peak as annealing temperature up to 450°C. From an overall perspective, *Q*_f_ amount was high and stable for the 450°C annealed samples deposited at temperature of 200°C, which is different with results elsewhere [6]. This may be due to different annealing atmospheres and annealing times or uncertainty evaluations. More fixed charge density means stronger field-effect passivation and repels minority/majority carriers away from surface to recombine.

As shown in Table 2, the amount of *Q*_f_ increased and *D*_it_ decreased by one magnitude after annealing. Effective minority carrier lifetime was high to 1,647.3 μs when deposition temperature was 200°C after annealing. Passivation quality was better for Al_2_O_3_ deposited at 200°C deposition temperature than at 100°C. The phenomenon was due to the deposition temperature of 100°C and was low to drive reaction Equation 1 as well [7]. After annealing at 450°C, the interface defect density of state between Al_2_O_3_ and Si is lower at deposition temperature of 200°C than at 100°C. That means the former can provide better surface passivation.

**Table 2:** **Surface fixed charge and the defect density of state vary with different heat treatments**

**PEALD**	***Q***_***f***_ **(cm** ^**−**^ **2)**	***D***_***it***_ **(eV** ^**−1**^ **cm** ^**−**^ **2)**	**Effective lifetime (μs)**
100°C as-deposited	−3.46 × 10^11^	7 × 10^12^	24.258
100°C annealing	−1.21 × 10^12^	6.1 × 10^11^	659.98
200°C as-deposited	−3.95 × 10^11^	1.64 × 10^12^	58.282
200°C annealing	−5.29 × 10^11^	1.2 × 10^11^	1,647.3

Annealing temperature is 450°C and measured by C-V method.

**Figure 4:**
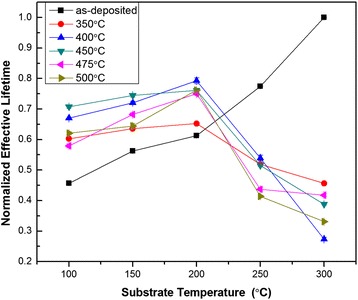
**Effective minority carrier lifetime variation.** At different deposition temperatures (substrate temperature, abscissa) and annealing temperature (ordinate).

As shown in Figure 4, before annealing, effective minority carrier lifetime increased with the increasing of deposition temperature (*T*_dep_). The reason for the increasing of *τ*_eff_ with the increasing of *T*_dep_ before annealing is that *D*_it_ decreased (chemical passivation quality increased) with *T*_dep_ increasing and *D*_it_ was high (little chemical passivation quality improvement would be dominant) before annealing. In Table 2, *D*_it_ decreased from 7 × 10^12^ to 1.64 × 10^11^ eV^−1^ cm^−2^ when *T*_dep_ increased from 100°C to 200°C before annealing. The microscopic mechanism is that H atoms have more chance (higher kinetic energy) at higher *T*_dep_ to diffuse to the terminal silicon dangling bonds and better chemical passivation is obtained. After annealing at variable temperatures, the highest *τ*_eff_ was obtained at the *T*_dep_ of 200°C. Comparing with the results of samples annealed at different temperatures, the optimized annealing temperature was 400°C to 450°C. The reason is that *Q*_f_ reached the biggest value when *T*_dep_ of Al_2_O_3_ was up to 200°C at the annealing temperature of 450°C (in Figure 3). Increasing of *Q*_f_ would enhance the field-effect passivation. After annealing, high *D*_it_ of PEALD Al_2_O_3_ because of UV irradiation in plasma process decreased significantly and chemical passivation became dominant. As reported [8], the defect density of states significantly decreased to 1 × 10^11^ eV^−1^ cm^−2^ after annealing, which is consistent with the result shown in Table 2. It is worth noting that *Q*_f_ of the samples as-deposited at 100°C and 200°C in Table 2 and Figure 3 do not match. The reason may due to the deviation of different measure methods.

**Figure 5:**
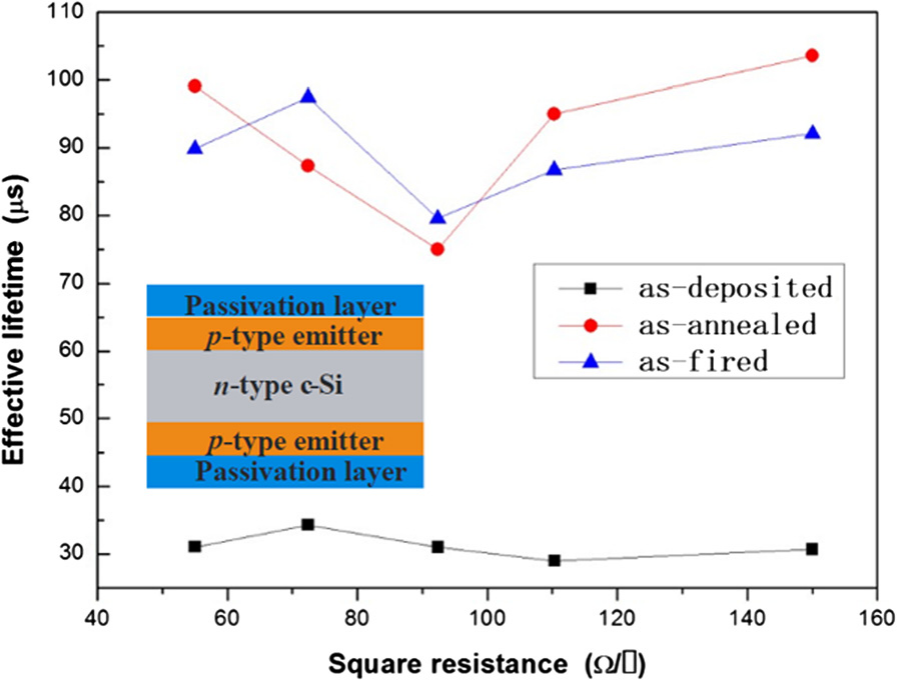
**Effective minority carrier lifetime varies on different Al diffusion concentrations.**

In order to investigate the dependence of Al_2_O_3_ passivation quality on doping concentration, n-type silicon was diffused double-side with Al to be P^+^ emitters, and passivation quality of PEALD alumina on P^+^ surface is shown in Figure 5. The Al_2_O_3_ films were deposited at 200°C. The thermal stability of Al_2_O_3_ films on the P^+^ layers were investigated by annealing at 450°C and firing with the same temperature curve as sintering Ag/Al contact during silicon solar cell process, in which the peak temperature was 800°C.

Before annealing, effective minority carrier lifetime increased with increased square resistance but began to decrease at the square resistance of 72 Ω/□. The phenomenon resulted from the combined action of the Al_2_O_3_/Si substrate and P^+^ emitter/Si substrate PN junction electron field. On well-passivated surfaces (after annealing), effective minority carrier lifetime increased with decreasing of doping concentration (increasing of square resistance) for decreasing SRH and Auger combination. However, *D*_it_ on as-deposited sample surface is high and causes worse passivation quality. Therefore, with square resistance increasing, PN junction field-effect passivation decays and effective minority carrier lifetime decreases. The result fitted well as reported previously [9]. After annealing at 450°C and for 10 min at ambient air, effective minority carrier lifetime decreased firstly and then increased with the increasing of square resistance. It was due to the interaction of the field-effect passivation decreasing and chemical passivation increasing (*D*_it_ decreased). After firing at about 800°C, the trend of effective minority carrier with P^+^ sheet resistance is similar with the curve of samples annealed at 450°C. Comparing with the result of samples annealed at 450°C, effective minority carrier lifetime changed slightly according to Figure 5 and disclosed the thermal stability of Al_2_O_3_ passivation via PEALD after firing at high temperature.

## Conclusions

In conclusion, we demonstrated that better uniformity of PEALD Al_2_O_3_ on 4′′ n-type silicon sample on the deviation of 0.7% was obtained when plasma power was changed to 45 W than 80 W. Growth rate also decreased with the deposition temperature increased. By analyzing surface fixed charge variation at different depositions and annealing temperatures, appropriate deposition temperature is 200°C, at this time, annealing temperature should be 450°C to obtain more fixed charge to enhance field-effect passivation. Annealing process was proven to be necessary to decrease surface defect density of state. Al diffusion P^+^ emitter on n-type silicon was passivated by PEALD Al_2_O_3_ films. After annealing, effective minority carrier lifetime increased to about 100 μs. Effective lifetime was high to about 100 μs when square resistance was about to 150 Ω/□ after annealing or firing.
